# Neurotransmitter-Mediated Signaling in Glioblastoma and Glial Tumors: Biology and Therapeutic Opportunities

**DOI:** 10.32604/or.2026.076088

**Published:** 2026-05-21

**Authors:** Pietro Tralongo, Mariagiovanna Ballato, Valeria Zuccalà, Vincenzo Fiorentino, Walter Giordano, Giovanna Casili, Fabiola Bellinghieri, Gerardo Caruso, Filippo Flavio Angileri, Guido Fadda, Maurizio Martini, Maria Caffo

**Affiliations:** 1 Department of Biomedical, Dental, Morphological and Functional Imaging Sciences, University of Messina, Messina, Italy; 2 Department of Human Pathology of Adults and Developmental Age “Gaetano Barresi”, Division of Pathology, University of Messina, Messina, Italy; 3 Department of Chemical, Biological, Pharmaceutical and Environmental Sciences, University of Messina, Messina, Italy; 4 School of Medicine and Surgery, University of Roma Tor Vergata, Rome, Italy; 5 Department of Biomedical and Dental Sciences and Morphofunctional Imaging, Unit of Neurosurgery, University of Messina, Messina, Italy

**Keywords:** Glioblastoma, cancer neuroscience, neuron–glioma synapse, neurotransmitter receptors, monoamines, drug repurposing, neurotransmitters, glioma, signaling

## Abstract

Glioblastoma (GB) is the most common primary malignant brain tumor of adulthood, and despite optimal safe resection and chemoradiation, it is still lethal. Neuroscience of cancer has shown that neuronal activities, as well as neurotransmitters, play an active role in the glioma microenvironment. This article aims to integrate the existing literature on the role of neurotransmitters and their receptors in glioblastoma, as well as other gliomas, highlighting areas of therapeutic intervention in the neuron-tumor interface. We will describe the neuro–glioma interface, including functional neuron–glioma synapses and activity-dependent tumor growth. We will also discuss major neurotransmitter systems involved in glioma pathobiology: glutamate, gamma aminobutyric acid, acetylcholine, dopamine, serotonin, norepinephrine, and other neurotransmitters. We will highlight that these neurotransmitter systems activate common intracellular signaling pathways that control tumor proliferation, invasion, metabolic reprogramming, immune suppression, therapy resistance, etc. In addition, some reports have found tumor-suppressing effects depending on the context. The involvement of neurotransmitter-driven signaling pathways represents a promising area of clinical potential in glioma pathobiology. In particular, focusing on key neurotransmitter systems with blood–brain barrier-permeable agents like alpha-amino-3-hydroxy-5-methyl-4-isoxazolepropionic acid (AMPA/X_c_^−^) system, Muscarinic acetylcholine receptor M3 (CHRM3), dopamine receptor D2, monoamine oxidase A, etc., may enhance drug-repurposing research as well as development of novel anti–neuron–glioma agents.

## 1 Introduction

Glioblastoma (GB) is the most common and aggressive primary malignant brain tumor in adults [1,2]. Its distinguishing features include rapid cell proliferation, diffuse infiltration into surrounding brain tissue, intense vascularization, and a profound resistance to treatment, resulting in a median survival of only 15 months despite an aggressive standard-of-care regimen of surgery, radiation, and chemotherapy [3,4,5]. The inherent difficulties in treating GB are exacerbated by the brain’s sensitive nature and the protective blood-brain barrier (BBB), which limits the administration of many therapeutic medicines [6]. The limited success of conventional medicines has prompted an extensive hunt for novel therapeutic vulnerabilities based on the disease’s specific biology.

Historically, glioma research has focused on the genetic and epigenetic changes that cause cancer. While this has provided essential insights, notably into key signaling pathways and mutations (e.g., in EGFR, PTEN, and IDH1/2), it is increasingly obvious that a tumor-centric perspective is insufficient to explain the full spectrum of GB malignancy [7,8]. The tumor microenvironment (TME) is now seen as an important player, consisting of a complex ecosystem of non-neoplastic cells that both influence and are influenced by the tumor [9].

The emerging discipline of cancer neuroscience has evolved to investigate the complex and reciprocal interaction between the neurological system and cancer [10,11,12]. This field has shown that neurons are not passive spectators, but rather actively co-opted by glioma cells to enhance their own development and survival [13,14,15]. This paradigm change is corroborated by bibliometric analyses, which demonstrate an increase in research linking neurotransmitters to cancer progression during the last two decades [16]. The complicated “cellular conversations” inside the glioblastoma ecosystem, which include not only tumor cells but also neurons, glia, and immune cells, are increasingly recognized as critical to its pathogenesis [17].

This review presents a comprehensive synthesis of current knowledge about how neurotransmitters and their signaling pathways contribute to the pathobiology of glioblastoma and other glial neoplasms. The purpose of this review is to offer an up-to-date and integrative overview of the role of neurotransmitter-mediated signaling in glioblastoma and other glial cell-derived tumors, with a particular emphasis on those processes that are most plausibly “druggable” in the near future. We will start by looking at the neuro-glioma interface, which is the anatomical and physiological basis for this communication. We will then thoroughly investigate the roles of specific neurotransmitter systems, drawing on a wide corpus of literature to provide a comprehensive picture of their influence. Compared with the existing reviews that focused on individual neurotransmitter axes, the current review highlights the implication of gliomas that coordinate multiple neurotransmitter systems simultaneously, and this review also underscores the translational aspects. Furthermore, this review also discusses the current potential targets for gliomas, such as repurposing strategies and pathway prioritization according to translational feasibility.

## 2 The Neuro-Glioma Interface

The brain’s complexity is derived from its network of trillions of synaptic connections. Surprising, new discoveries have revealed that gliomas actively integrate into these networks, creating a functional, although malignant, component of the brain’s circuitry [13,14].

### 2.1 Neuron-Glioma Synapse

Early studies suggested this integration by showing that glioma cells and cell lines express a diverse range of functional neurotransmitter receptors, including glutamate, GABA, acetylcholine, and others [18,19,20]. Cultured human glioma cells were demonstrated to respond to these neuroligands with intracellular calcium signals, indicating that they were able to “listen” to neural transmission [21]. It is now clear that this communication is not purely paracrine, but takes place via specialized, synapse-like junctions.

**Figure 1:**
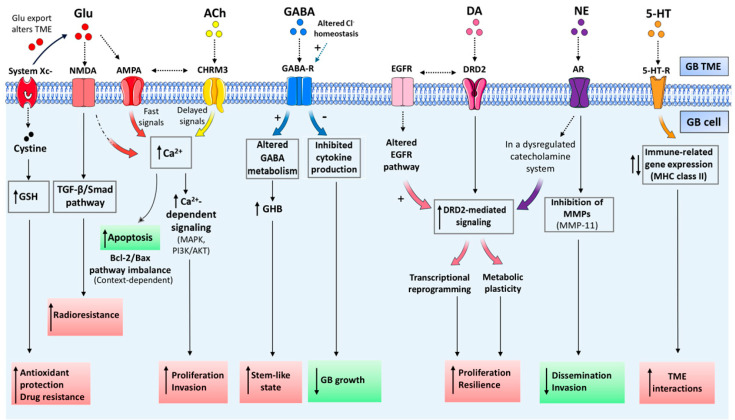
Neurotransmitter-driven networks in GB. This conceptual figure illustrates the principal neurotransmitter systems, including Glutamate, Acetylcholine, GABA, and Dopamine, together with key neuromodulators, such as Serotonin and Norepinephrine, that actively shape GB behavior, highlightinging the downstream signaling pathways and their effects on tumor progression. Pro-tumorigenic and anti-tumorigenic effects are indicated in red and green, respectively. The schematic further outlines the existence of a complex and dynamic crosstalk between neurotransmitter and oncogenic pathways, as well as their convergence on common downstream mediators, suggesting that the overall impact on GB reflects the integration of multiple signals. Abbreviations: GB, Glioblastoma; Glu, Glutamate; ACh, Acetylcholine; GABA, Gamma-aminobutyric acid; EGFR, Epidermal growth factor receptor; DA, Dopamine; NE, Norepinephrine; 5-HT, Serotonin; TME, Tumor microenvironment; NMDA, N-methyl-D-aspartate; AMPA, alpha-amino-3-hydroxy-5-methyl-4-isoxazolepropionic acid; CHRM3, Muscarinic acetylcholine receptor M3; GABA-R, Gamma-aminobutyric acid receptor; DRD2, Dopamine receptor 2; AR, Androgen receptor; 5-HT-R, Serotonin receptor; GSH, Glutathione; TGF-beta, Transforming growth factor beta; BCL2, B-cell lymphoma 2; Bax, BCL2-associated X protein; Ca^2^^+^, calcium; MAPK, Mitogen-activated protein kinase; PI3K, Phosphoinositide 3-kinase; AKT, Protein kinase B; GHB, gamma-hydroxybutyrate; MMPs, Matrix metalloproteinases; MHC, Major histocompatibility complex. The figure was created using Servier Medical Art (https://smart.servier.com, accessed on 30 October 2025) and NIH BioArt (https://bioart.niaid.nih.gov, accessed on 30 October 2025).

Researchers used high-resolution electron microscopy to reveal direct, bona fide synapses between presynaptic neuronal axons and postsynaptic glioma cell membranes [14,22] (Fig. 1). These neuron-glioma synapses have the characteristics of canonical interneuronal synapses, such as vesicle-filled axonal boutons and postsynaptic densities, which provide a physical substrate for direct, fast, and activity-dependent signal transmission [14]. The protein makeup of these interfaces is also being researched, with synaptic proteins such as bassoon, DLG4, and HOMER1 being studied in glioma [23].

To trace the origins of these inputs, researchers used sophisticated techniques such as retrograde monosynaptic rabies virus tracking [24]. Yang et al. used this approach to generate a brain-wide connectome map for xenografted human GB, revealing a consistent organizational logic: tumors receive dense local inputs, primarily glutamatergic, as well as diverse, long-range inputs from various subcortical neuromodulatory systems, including cholinergic neurons from the basal forebrain [2]. Other investigations employing similar approaches have verified this extensive neuroanatomical integration, exhibiting different electrical features of the glioma-innervating neurons [25,26,27,28]. This integration is a critical component of glioma biology, allowing the hijacking of the brain’s signaling apparatus. To support this integration notion, certain investigations have shown that glioblastoma cells can be reprogrammed or forced to develop into neurons or oligodendrocytic cells, blurring the distinction between neoplastic and neural cell identities [29,30,31]. Communication at this contact is also mediated by gap junctions, with connexins 30, 36, and 43 involved in both normal brain function and brain malignancies [32].

### 2.2 Activity-Dependent Glioma Progression

The structural integration of gliomas is accompanied by significant functional effects. Neuronal activity is no longer viewed as simply background noise, but rather as a powerful driver of tumor growth [13]. Optogenetic activation of cortical neurons surrounding a glioma xenograft was found to greatly boost tumor cell proliferation, indicating a clear causal relationship between brain activity and glioma growth [13]. This effect is mediated, in part, by the activity-dependent production of molecules that promote tumor growth, such as neuroligin-3 (NLGN3) and brain-derived neurotrophic factor (BDNF) [13,33]. Furthermore, neuronal activity from remote brain regions can promote glioma growth by releasing signaling proteins such as semaphorin 4F (SEMA4F) [34].

**Figure 2:**
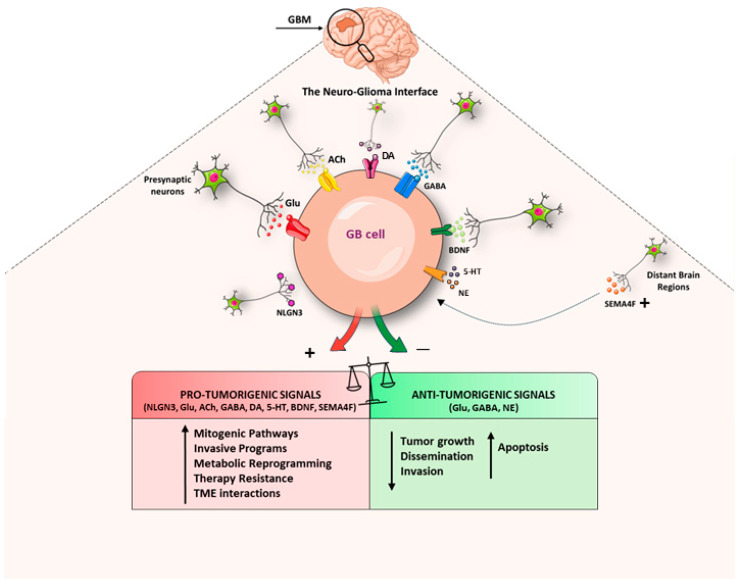
Schematic representation of Neuron-Glioma Synapses. GB cells integrate into normal neuronal circuits, manipulating them to shape a tumor-supportive ecosystem. Through the expression of specific receptors, they sense neurotransmitters such as Glutamate, GABA, and Acetylcholine as well as neurotrophic factors like BDNF, triggering intracellular pathways that promote tumor proliferation, invasiveness, resilience, and metabolic adaptation. Abbreviations: GB, Glioblastoma; Glu, Glutamate; ACh, Acetylcholine; GABA, Gamma-aminobutyric acid; DA, Dopamine; 5-HT, Serotonin; NLGN3, Neuroligin-3; BDNF, Brain-derived neurotrophic factor; SEMA4F, Semaphorin 4F; NE, Norepinephrine. The figure was created using Servier Medical Art (https://smart.servier.com, accessed on 15 January 2026) and NIH BioArt (https://bioart.niaid.nih.gov, accessed on 15 January 2026).

At the synaptic level, this functional integration manifests as neurotransmitter-driven electrical activity in glioma cells. Action potentials in presynaptic neurons cause the release of glutamate, which activates AMPA receptors on glioma cells, resulting in depolarization and calcium influx, which promotes proliferation and invasion [14]. This generates a vicious feedback loop, as gliomas can cause neuronal hyperexcitability and seizures by releasing excess glutamate, which then drives tumor growth [14,35]. Indeed, the relationship between neurotransmitters and glioma-associated seizures is the subject of much clinical and scientific study [16,35,36]. This malignant feedback loop emphasizes gliomas’ profound and damaging integration into the brain’s functioning networks. In regard to tumor-promotive signals, while the synaptic release of glutamate and neuromodulators from active neuronal networks is the most important contributing factor, glioma cells are additionally capable of modifying the landscape of neurotransmitters. In fact, the export of glutamate via the system X_c_^−^ is an exemplary pathway, as it participates simultaneously in redox balance by importing cystine. An additional pathway, which is emerging as important but for which results so far are limited to the literature on glioblastomas and monoamine neurotransmission, is the possible utilization of the molecular components of the monoamine system by glioblastoma cells (Fig. 2).

## 3 Major Neurotransmitters as Regulators of Glioma Pathobiology

The broad array of neurotransmitter receptors expressed by glioma cells gives them the ability to respond to and control the brain’s chemical landscape. Here, we present a detailed overview of the key neurotransmitter systems and their functions in glioma (Table 1).

**Table 1:** Major neurotransmitter systems, receptors, downstream signaling, and translational implications in glioblastoma.

Neurotransmitter	Receptor	Pathogenetic Mechanisms and Pro-/Anti-Tumor Roles in GB	Translational Implications
Glutamate (Glu)	Ionotropic receptors: α-amino-3-hydroxy-5-methyl-4-isoxazolepropionic acid (AMPA) *N*-methyl-D-aspartate (NMDA) Kainate Metabotropic receptors: mGluRs	Pro-tumor: - AMPA-mediated Ca^2+^ influx promotes GB proliferation and invasion - NMDA receptor signaling modulates the Transforming Growth Factor-beta (TGF-β)/Smad pathway inducing radioresistance - Glu released from GBM cells via system X_c_^−^ supports excitotoxicity, tumor growth, chemoresistance, and oxidative stress - 2-HG production in Isocitrate dehydrogenase (IDH)-mutant gliomas alters Glu metabolism, supporting tumor growth Anti-tumor (context-dependent): - Glu can induce apoptosis of GB cells via B-cell lymphoma 2/BCL2 associated X protein (Bcl-2/Bax) modulation	- Targeting system X_c_^−^ to increase GB vulnerability to treatments - Monitoring metabolic changes as potential diagnostic, prognostic, and predictive biomarkers
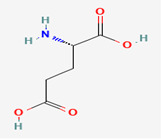			
γ-Aminobutyric acid (GABA)	GABA-A	Pro-tumor: - Altered chloride gradient via SLC12A5 in DMGs drives tumor progression - Altered GABA metabolism, producing gamma-hydroxybutyrate, supports a proliferative, stem-like state Anti-tumor: - GABA suppresses tumor growth by inhibiting cytokine release from GB cells	- GABA-A receptor subunit expression as diagnostic and prognostic biomarker - SLC12A5 as predictor of GABAergic response in GB
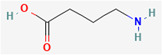			
Acetylcholine (ACh)	Cholinergic receptor muscarinic 3 (CHRM3), nAChR	Pro-tumor: - Activation of cholinergic receptors promotes proliferation, invasion, and GSC maintenance	- CHRM3 blockade prevents GB progression - Potential repositioning of anticholinergic drugs
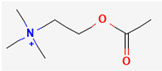			
Dopamine	Dopamine receptor D2 (DRD2)	Pro-tumor: - Epidermal growth factor receptor (EGFR) pathway-dependent DRD2 overexpression drives transcriptomic remodeling and metabolic plasticity in GBM cells, stimulating tumor proliferation and therapy resistance	- Potential Repurposing of DRD2 antagonists for GB treatment
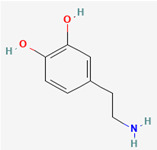			
Serotonin (5-HT)	Serotonin/5-hydroxytryptamine (5-HT) transporters	Pro-tumor: - Modulating MHC-II and other immune gene expression, 5-HT supports neuron-immune interactions in TME	- Target-specific imaging through the serotonin transporter expression - MAO-A inhibition as a prospective therapeutic approach
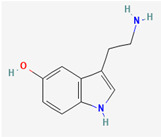			
Norepinephrine (NE)	Adrenergic receptors	Pro-tumor: - When part of a dysregulated catecholamine system: NE is linked to GBM development Anti-tumor: - Inhibition of MMP-11 reduces *in vitro* dissemination and invasion of GBM cells	- Targeting Monoamine oxidase A (MAO-A) as a potential therapeutic strategy
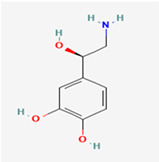			

**Note:** This table summarizes the key neurotransmitters variously implicated in GBM biology, highlighting the tumor’s multifaceted nature. It details specific receptors, key molecular mechanisms and their pro- and anti-tumorigenic roles in glioblastoma (GB), as well as potential future translational implications. Abbreviations: GB, glioblastoma; mGluRs, metabotropic glutamate receptors; 2-HG, 2-hydroxyglutarate; SLC12A5, solute carrier family 12 member 5; DMGs, diffuse midline gliomas; GSC, glioma-stem cell; TME, tumor microenvironment; MMP-11, matrix metalloproteinase-11. 2D chemical structures were obtained from PubChem (https://pubchem.ncbi.nlm.nih.gov; accessed 30 October 2025).

### 3.1 Glutamate

As the main excitatory neurotransmitter in the central nervous system (CNS), glutamate is possibly the most researched neurotransmitter in the setting of glioma, where it plays a central and diverse pro-tumorigenic role [35,37,38].

#### 3.1.1 Glutamate Receptors and Signaling in Glioma

Glioma cells express the entire range of ionotropic glutamate receptors (AMPA, NMDA, and kainate) and metabotropic glutamate receptors (mGluRs) [19,39,40]. Synaptically-released glutamate activates ionotropic AMPA receptors on the glioma cell membrane, causing membrane depolarization and a fast influx of calcium [14,41]. This calcium signaling serves as a hub for a number of downstream pathways that regulate proliferation and invasion [42,43]. AMPA receptors have long been studied, and their significance in glioma adds to their complex biology [44]. Research indicates that NMDA receptors may influence TGF-β/Smad pathways in response to radiation, potentially leading to therapeutic resistance [45]. The glutamate released by glioma cells can cause excitotoxic death of nearby neurons, allowing the tumor to grow and contributing to GB’s necrotic core [38,46]. The Bcl-2/Bax pathway has also been implicated in the glutamate-induced apoptosis of glioma cells [47].

#### 3.1.2 The Cystine/Glutamate Antiporter (System X_c_^−^)

In addition to responding to neuronal glutamate, glioma cells actively release their own glutamate via system X_c_^−^, a highly expressed cystine/glutamate antiporter encoded by SLC7A11 [48,49]. This transporter imports cystine, which is required for the formation of the antioxidant glutathione (GSH), shielding the tumor from oxidative stress and increasing chemoresistance [49,50]. The concomitant export of glutamate alters the peritumoral milieu, contributing to excitotoxicity, neuronal hyperexcitability, and seizures [48,49,51]. High system X_c_^−^ expression is associated with glioma-related epilepsy and acts as an independent biomarker for seizures at diagnosis [51]. As a result, targeting this antiporter is being investigated as an approach to sensitize glioma cells to therapy [49,50].

#### 3.1.3 Glutamate Metabolism and IDH Mutations

Gliomas, particularly those with mutations in IDH1 or IDH2, undergo extensive metabolic reprogramming [52,53]. IDH-mutant gliomas create 2-hydroxyglutarate (2-HG), an oncometabolite that might affect glutamate metabolism [54]. Human-specific enzymes, such as GLUD2, which is involved in glutamate metabolism, have been found to accelerate the formation of IDH1-mutant glioma [55]. These metabolic changes are associated not only to cancer, but also to clinical symptoms such as seizures [56]. Advanced imaging techniques, such as MR spectroscopy, can detect these metabolic changes *in vivo*, revealing variations in glutamate, glycine, and other metabolites that can be used as biomarkers for tumor type and development [57,58,59]. For example, the use of lactate as a predictor of survival and responsiveness to radiation therapy has been investigated [60,61].

### 3.2 GABA

GABA, the brain’s primary inhibitory neurotransmitter, plays a more nuanced and context-dependent role in glioma development than glutamate [62]. While some studies have suggested that GABA has a tumor-suppressive role by blocking cytokine production from glioma cells [63], others have found that altering GABA signaling can influence the formation of glioblastoma spheroids [64]. The expression of GABA-A receptor subunits is associated with tumor histology and clinical prognosis, demonstrating their clinical importance [65]. However, the most striking finding has been GABA’s paradoxical excitatory and pro-tumorigenic involvement in some glioma subtypes. In diffuse midline gliomas (DMGs), GABAergic synaptic transmission increases tumor development [66]. This is owing to an altered chloride gradient in tumor cells, resulting in GABA-A receptor activation that is depolarizing rather than hyperpolarizing [66]. The expression of chloride transporters such as SLC12A5 (KCC2) is thus an important predictor of the GABAergic response in glioblastoma [67]. Furthermore, glioma cells can have altered GABA metabolism, with the generation of gamma-hydroxybutyrate (GHB) being a key factor in maintaining a proliferative, stem-like state [68]. These findings indicate extraordinary adaptability in how gliomas can exploit even inhibitory signals to their benefit.

### 3.3 Acetylcholine

The cholinergic system, a fundamental regulator of cortical arousal and plasticity, has recently been identified as a critical long-term modulator of glioma growth [2,69]. Yang et al. describe how cholinergic neurons in the basal forebrain create functional synapses with GB cells, and the release of acetylcholine (ACh) at these junctions stimulates tumor proliferation and invasion [2]. This impact is predominantly mediated by the metabotropic muscarinic M3 receptor (CHRM3) [2]. Other investigations have confirmed the pro-tumorigenic effect of muscarinic signaling in GSCs, revealing that blocking these receptors prevents tumor growth [70,71]. Beyond muscarinic receptors, nicotinic ACh receptors are expressed on glioma cells and can be regulated by a variety of drugs, with certain neurotoxic inhibitors promoting growth [72]. The clinical relevance is highlighted by the discovery that systemically administered drugs can have an effect; for example, neuromuscular blocking agents such as atracurium can promote astroglial differentiation and deplete the GSC pool [73], whereas the anesthetic midazolam can epigenetically influence cholinesterase gene expression [74]. It has also been revealed that human glioblastoma cells can differentiate into cholinergic neuron phenotypes, demonstrating the tumor cells’ flexibility [28]. The identification of the ACh-CHRM3 axis as a driver of GB growth has important therapeutic implications, indicating the possibility of repurposing anticholinergic drugs [2].

### 3.4 Dopamine

Dopamine signaling has a role in a variety of neurological activities, and dysregulation is key to disorders such as Parkinson’s and schizophrenia. In glioblastoma, the dopamine system, namely the D2 receptor (DRD2), has emerged as a difficult but potential therapeutic target [75,76].

DRD2 is overexpressed in GB, particularly in GSCs, and activation has been demonstrated to accelerate tumor growth [77,78]. A genome-wide screen identified DRD2 as a major mitogenic signaling hub that interacts with the epidermal growth factor receptor (EGFR) pathway [79,80]. DRD2 activation causes substantial transcriptome and metabolic plasticity in GB cells, which contributes to aggressive behavior and treatment resistance [81,82]. Chronic stress, a known modulator of dopamine signaling, can hasten GB growth via a DRD2-dependent axis [83]. Glioma formation can also affect striatal dopaminergic function in the host brain [84].

This pro-tumorigenic activity makes DRD2 an appealing target. Many antipsychotics are DRD2 antagonists that can pass the blood-brain barrier. Preclinical investigations have demonstrated that medicines such as thioridazine, haloperidol, and pimozide can decrease glioma growth [80,85]. This has sparked a great interest in repurposing these psychiatric medications for GB treatment [85]. Dopamine signaling appears to alter glioma cells’ physical features, which contribute to their spheroid forming behavior [86]. The effect of dopaminergic action on the glioblastoma niche is a current research topic [87].

### 3.5 Monoamines: Serotonin and Norepinephrine (NE)

Other monoamines contribute to the complex biology of GB [75]. Serotonin (5-HT) has been studied for its possible application in targeted imaging, as glioma cells produce serotonin transporters [88]. Serotonin can also influence the expression of immune genes such as MHC class II on glioma cells, implying a role in neuro-immune interactions at the TME [89]. The involvement of the serotonergic system in other brain diseases raises concerns regarding its function in the neurological and behavioral symptoms associated with glioma [90]. More recent work has continued to support the relevance of the serotonergic axis in GB by demonstrating expression and distribution of key serotoninergic system proteins in glioblastoma samples and datasets, with subtype-specific patterns reported across tumors. This supports the concept that serotonin-related machinery may contribute to intratumoral heterogeneity and could help explain patient-to-patient variability in neuromodulator responsiveness. From a translational perspective, the presence of serotonin transport and receptor components strengthens the rationale for serotonin-pathway imaging strategies and invites further investigation into whether serotonergic signaling participates in immune remodeling or adaptive resistance programs [91].

NE appears to play a contrasting, perhaps tumor-suppressive role. One study discovered that NE suppresses the migration and invasion of glioblastoma cells *in vitro*, perhaps by inhibiting MMP-11 [92]. However, the larger catecholamine system, which includes both dopamine and norepinephrine, has been linked to glioblastoma formation, indicating a complicated interaction [93,94].

Monoamine oxidases (MAOs) regulate monoamine metabolism. Monoamine oxidase A (MAO-A) is overexpressed in gliomas, and inhibiting it has been demonstrated to slow glioma growth, making it a prospective therapeutic target [95,96]. Glucocorticoids and androgens can promote MAO-A expression, which connects stress and hormonal signaling to glioma biology [97].

### 3.6 Other Neurotransmitters and Neuromodulators

Chemical communication within the glioma TME expands beyond the traditional neurotransmitters to include a variety of additional signaling chemicals.

#### 3.6.1 Neuropeptides

Glioma cells have receptors for several neuropeptides. Substance P’s receptor, the neurokinin-1 receptor (NK-1R), is thought to be a possible target for GB treatment [98]. Neuropeptide Y (NPY) Y2 receptors are present and functional in glioblastoma cell lines [99]. The secretin/PACAP/VIP peptide families also functions in the CNS, and neuroleptic medications regulate their receptors in glioma cells [100,101]. Some glioblastoma cell lines also express TRH and TRH-like peptides [102].

#### 3.6.2 Purines

ATP released into the TME can function as a neurotransmitter. Glioma cells respond to ATP by an increase in intracellular calcium and glutamate release, resulting in a feed-forward cycle of excitation [103]. Adenosine signaling is also an important regulator of the glioma microenvironment and its interaction with reactive astrocytes [104].

#### 3.6.3 Endocannabinoids

The endocannabinoid system, which includes signaling molecules such as anandamide, has been found to have anti-proliferative effect against glioblastoma cells *in vitro* [105,106,107].

#### 3.6.4 Amino Acids

Aside from glutamate, additional amino acids function as neurotransmitters or neuromodulators. Glycine has been identified as a biomarker in brain malignancies via high-resolution MR spectroscopy [59,60]. In glioblastoma cells, nitric oxide regulates D-serine, an NMDA receptor co-agonist, and its synthesis enzyme, serine racemase [108]. Proline metabolism has also been thoroughly investigated for its function in malignant gliomas [109].

#### 3.6.5 Nitric Oxide (NO)

This gaseous signaling molecule has been linked to malignant glioma growth and the modulation of serine racemase activity [108,110].

## 4 Crosstalk and Integrated Signaling Networks

Neurotransmitters’ influence on glioma is not determined by a single, linear process. Instead, these signals are incorporated into complex networks with substantial crosstalk and convergence, which together dictate the tumor’s behavior.

Yang et al. give a compelling example of this integration, demonstrating how cholinergic and glutamatergic signals interact to augment glioma calcium transients [2]. While co-activation of both routes increased the size of the calcium signal, it had different impacts on the temporal dynamics of transcriptional control, demonstrating that they are not redundant [2]. This shows that gliomas use both fast, ionotropic signals (glutamate) and delayed, metabotropic neuromodulatory signals (ACh) to fine-tune their proliferative and invasive programs on various time scales.

Another crucial area of integration is between neurotransmitter signaling and conventional carcinogenic pathways. The discovery that dopamine receptor D2 (DRD2) signaling interacts with the EGFR pathway to enhance mitogenesis is a prime example [79,80]. This reveals that neurotransmitter inputs do not work in isolation, but can increase or affect the output of key cancer-causing pathways. 

The balance of excitatory and inhibitory inputs, such as glutamate and GABA, is also thought to be a key factor in determining the overall state of the neuro-glioma network. As demonstrated in DMGs, switching GABAergic transmission from inhibitory to excitatory would significantly alter this balance, resulting in a very pro-tumorigenic environment [66]. The varying effects of neurotransmitters on different glioblastoma subtypes highlight the importance of a tailored understanding of the neuro-glioma chemical environment [111].

Acute signaling, however, is not the only mode where neurotransmitters play a role, and they have also been implicated in the ability to regulate the identity of tumor cells over the long term. Calcium transients mediated by the AMPA/NMDA, and possibly monoaminergic signaling initiated by GPCRs, may regulate chromatin-bound transcriptional states via Ca^2+^ signaling nodes, or result in the remodeling of gene transcription via second messenger cascades such as cAMP/PKA/CREB. At the same time, the effects of neurotransmitter signaling and metabolic reprogramming may also regulate the levels of the substrates of chromatin remodeling, such as acetyl-CoA and SAM, and thus regulate histone acetylation and methylation levels. These considerations become highly relevant when focusing on the glioma stem-like cells, where chromatin remodeling mediated by neurotransmitter signaling may be pivotal for sustaining proliferation states and contributing to the acquisition of therapy-resistant states and possibly to the bias of lineage plasticity, and for the emerging role of neurotransmitters and epigenetic regulation in glioblastoma [112].

The functional role of neurotransmitter networks is presumably context-dependent across GB subtypes. By way of explanation, IDH-mutant gliomas have specific metabolic profiles and oncometabolite-mediated chromatin regulation mechanisms that may alter glutamate network function and neuronal excitation patterns compared to IDH-wildtype GBs. Likewise, the difference between the proneural and mesenchymal phases may be associated with variable degrees of synaptic integration, neuromodulator sensitivity, and intercellular coupling. In this background scenario, gap junction-mediated mechanisms may contribute to maintaining malignancy through specific networks associated with Connexin43, recently proposed as an unconventional phenotypic stability factor in glioblastomas by promoting a hybrid epithelial/mesenchymal phenotype [113].

## 5 Therapeutic Implications and Future Directions

Future research must go beyond researching individual neurotransmitter systems and take a more holistic, systems-level approach to understanding how the tumor integrates these various chemical signals to orchestrate its malignant growth. The varying effects of neurotransmitters on different glioblastoma subtypes highlight the importance of a tailored understanding of the neuro-glioma chemical environment [111] (Table 2).

**Table 2:** Overview of representative preclinical and clinical studies exploring neurotransmitter-related targets and drug repurposing in GBM.

	Author/Year, References	Study Type	Neurotransmitter/Target	Intervention/Drug	Model/Population	Main Findings	Therapeutic Relevance
1	Yang et al., 2025 [2]	Preclinical (xenograft, optogenetics)	Acetylcholine/CHRM3	Cholinergic activation; muscarinic receptor antagonists	Mouse xenograft model of GB	Activation of basal forebrain cholinergic neurons enhances glioma proliferation and invasion	Identified CHRM3 as a therapeutic target; supports repurposing of anticholinergic drugs (e.g., scopolamine)
2	Venkataramani et al., 2019 [14]	Preclinical (EM imaging, *in vivo*)	Glutamate/AMPA receptors	AMPA receptor antagonist (e.g., perampanel)	Human GB xenografts	Discovery of bona fide neuron–glioma synapses transmitting glutamatergic signals promoting proliferation	Provides rationale for AMPA antagonists such as perampanel
3	GLUGLIO Trial (Phase Ib/II, ongoing) [114,115]	Clinical trial	Glutamate signaling	Glutamate signaling inhibitors + standard chemoradiotherapy	Newly diagnosed GB patients	Evaluating safety and efficacy of glutamate blockade in combination therapy	First clinical translation of glutamatergic targeting in GB
4	Various (Haloperidol, Thioridazine studies) [80,85]	Preclinical/Pharmacological	Dopamine/DRD2	DRD2 antagonists (antipsychotics)	GB cell lines and animal models	DRD2 blockade reduces proliferation and therapy resistance	Strong rationale for repurposing antipsychotics as GB therapeutics
5	Rasagiline studies [116]	Preclinical	Monoamines/MAO-A	MAO-A inhibitor (rasagiline)	Glioma cell lines	MAO-A inhibition slows glioma growth	Potential for repurposing Parkinson’s drugs targeting MAO-A

**Abb:** CHRM3: Cholinergic receptor muscarinic 3.

### 5.1 Drug Repurposing

The most urgent therapeutic option is to repurpose medications that have already been licensed for neurological or psychiatric conditions [85].

**Figure 3:**
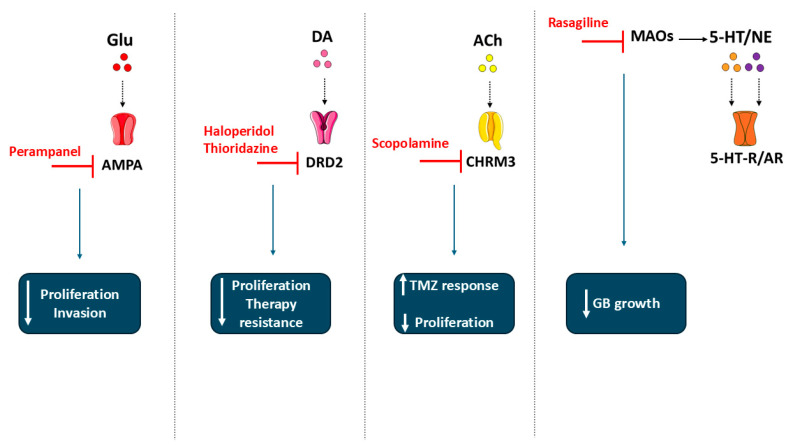
A therapeutic strategy map connecting pharmacological agents currently explored for drug repurposing to their corresponding molecular targets within neurotransmitter signaling pathways in GB, providing a preliminary overview of possible therapeutic intervention points and their potential anti-tumorigenic effects. Abbreviations: GB, Glioblastoma; Glu, Glutamate; AMPA, alpha-amino-3-hydroxy-5-methyl-4-isoxazolepropionic acid; DA, Dopamine; DRD2, Dopamine receptor 2; ACh, Acetylcholine; CHRM3, Muscarinic acetylcholine receptor M3; MAOs, Monoamine oxidases; 5-HT, Serotonin; 5-HT-R, Serotonin receptor; NE, Norepinephrine; AR, Androgen receptor. The figure was created using Servier Medical Art (https://smart.servier.com, accessed on 30 October 2025) and NIH BioArt (https://bioart.niaid.nih.gov, accessed on 30 October 2025).

From the near-term clinical viewpoint, those most actionable pathways are represented by glutamate/AMPA/system X_c_^−^ signaling given seizure overlap and existing anti-glutamatergic drugs; CHRM3-driven cholinergic inputs; and DRD2 signaling leveraging BBB-permeable antagonists, besides MAO-A inhibition. Each is supported by repurposable neuroactive compounds and emerging translational evidence (Fig. 3).

#### 5.1.1 Glutamatergic Antagonists

Given glutamate’s central role, drugs targeting its receptors are of high interest [37]. The non-competitive AMPA receptor antagonist perampanel, used for epilepsy, has shown preclinical efficacy [14,22]. The GLUGLIO phase Ib/II trial is currently evaluating glutamate signaling inhibitors in combination with standard chemoradiotherapy in newly diagnosed GB, a landmark step in translating this science to the clinic [114,115].

#### 5.1.2 Dopaminergic Modulators

DRD2 antagonists, such as the antipsychotics haloperidol and thioridazine, have demonstrated potent anti-glioma activity in preclinical models and are being investigated for repurposing [80,85].

#### 5.1.3 Cholinergic Antagonists

The finding of the pro-tumorigenic role of the ACh-CHRM3 axis makes mAChR antagonists such as scopolamine promising candidates for repurposing, especially as they showed an additive effect with TMZ [2].

#### 5.1.4 MAO Inhibitors

Monoamine oxidase A (MAO-A) inhibitors, such as the Parkinson’s drug rasagiline, have been demonstrated to slow glioma growth, opening up new opportunities for repurposing [95,96,116,117].

### 5.2 Novel Therapeutic Strategies

Beyond repurposing, a better knowledge of the neuro-glioma relationship will drive the creation of new treatments. These could include highly specific receptor antagonists, tiny compounds that disrupt neuron-glioma connections, or even gene treatments that suppress critical receptor genes via RNA interference [118]. Another interesting approach is to use aptamers as molecular recognition elements for CNS diagnostics and therapies [119]. Advanced drug delivery strategies, such as convection-enhanced delivery, may be required to cross the BBB and effectively deliver these medicines to the tumor location [6].

### 5.3 Challenges and Future Outlook

Transforming these intriguing ideas into clinical success necessitates overcoming considerable challenges. The BBB remains a significant challenge for several potential medicines [120]. GB’s considerable inter- and intratumoral variability makes a “one-size-fits-all” strategy difficult to succeed [121,122]. A future technique could include assessing a patient’s tumor for a unique neurotransmitter receptor expression signature to guide tailored therapy. Finally, the potential risk of on-target neurological side effects from systemically altering neurotransmitter systems is a major worry that must be addressed through targeted delivery, careful dosing, or the discovery of therapies with greater tumor selectivity.

Key limitations include the predominance of preclinical models, incomplete mapping of receptor expression across the GB subtypes, and difficulty in distinguishing neuronal versus tumor-derived neurotransmitter effects within patient tissues. Moreover, BBB penetration and on-target CNS toxicity remain central constraints that underscore the need for biomarker-guided stratification and targeted delivery strategies.

The future of glioma therapy will most likely involve multi-pronged strategies that combine traditional cytotoxic treatments with techniques that alter the tumor’s permissive microenvironment. This could include combination therapy that target both glutamatergic and cholinergic signaling [2], as well as combining a DRD2 antagonist with EGFR inhibitors. The ultimate goal is to change the brain’s permissive, supporting environment into one that is hostile to tumor formation.

## 6 Conclusion

The study of neurotransmitters in glioblastoma has revealed a new layer of cancer biology, reinterpreting this lethal condition as a brain circuit problem. Gliomas are more than just collections of cancerous cells; they are intricately intertwined into the brain’s framework, listening to and altering the language of neurotransmitters. From glutamate’s persistent excitatory drive to acetylcholine’s delicate, long-range regulation and dopamine’s complex interplay, these chemical messengers play critical roles in cancer.

This new perspective offers both a deeper awareness for the complexities of glioblastoma and further therapeutical possibilities to the patients. The discovery of these neuro-glioma communication channels has revealed numerous new treatment targets. The capacity to reuse previously approved medications expedites clinical translation, while ongoing research into the molecular basis of this interface promises an exciting future of tailored therapeutics. By learning to intercept the communication between neurons and glioma cells, we may be able to silence those signals that drive this disease and rewrite the future for glioblastoma patients.

## Data Availability

Not applicable.

## References

[1] Jung E , Alfonso J , Osswald M , Monyer H , Wick W , Winkler F . Emerging intersections between neuroscience and glioma biology. Nat Neurosci. 2019; 22( 12): 1951– 60. doi:10.1038/s41593-019-0540-y. 31719671

[2] Yang Y , Yang C , Chen X , Jiang Y , Lei X , Ma K , et al. Long-range cholinergic input promotes glioblastoma progression. Cancer Cell. 2025; 43( 11): 2089– 105.e10. doi:10.1016/j.ccell.2025.07.024. 40829591

[3] Ostrom QT , Price M , Neff C , Cioffi G , Waite KA , Kruchko C , et al. CBTRUS statistical report: Primary brain and other central nervous system tumors diagnosed in the United States in 2016–2020. Neuro Oncol. 2023; 25( Suppl 4): iv1– 99. doi:10.1093/neuonc/noad149. 37793125 PMC10550277

[4] Sabouri M , Dogonchi AF , Shafiei M , Tehrani DS . Survival rate of patient with glioblastoma: A population-based study. Egypt J Neurosurg. 2024; 39( 1): 42. doi:10.1186/s41984-024-00294-5.

[5] Fekete B , Werlenius K , Tisell M , Pivodic A , Smits A , Jakola AS , et al. What predicts survival in glioblastoma? A population-based study of changes in clinical management and outcome. Front Surg. 2023; 10: 1249366. doi:10.3389/fsurg.2023.1249366. 37711136 PMC10498299

[6] Bota DA , Di K , Keator DB , Bota RG , Hoffmann M , Dumitru CD , et al. Abstract 4733: Human functional brain imaging data support preclinical and clinical evidence that marizomib crosses the blood-brain barrier (BBB) to inhibit proteasome activity in the brain. Cancer Res. 2019; 79( Suppl 13): 4733. doi:10.1158/1538-7445.AM2019-4733.

[7] Bao H , Ren P , Yi L , Lv Z , Ding W , Li C , et al. New insights into glioma frequency maps: From genetic and transcriptomic correlate to survival prediction. Int J Cancer. 2023; 152( 5): 998– 1012. doi:10.1002/ijc.34336. 36305649 PMC10100131

[8] Wang A , Zhang G . Differential gene expression analysis in glioblastoma cells and normal human brain cells based on GEO database. Oncol Lett. 2017; 14( 5): 6040– 4. doi:10.3892/ol.2017.6922. 29113243 PMC5661398

[9] Sharma P , Aaroe A , Liang J , Puduvalli VK . Tumor microenvironment in glioblastoma: Current and emerging concepts. Neuro Oncol Adv. 2023; 5: vdad009. doi:10.1093/noajnl/vdad009. PMC10034917 36968288

[10] Monje M , Borniger JC , D’Silva NJ , Deneen B , Dirks PB , Fattahi F , et al. Roadmap for the emerging field of cancer neuroscience. Cell. 2020; 181( 2): 219– 22. doi:10.1016/j.cell.2020.03.034. 32302564 PMC7286095

[11] Winkler F , Venkatesh HS , Amit M , Batchelor T , Demir IE , Deneen B , et al. Cancer neuroscience: State of the field, emerging directions. Cell. 2023; 186( 8): 1689– 707. doi:10.1016/j.cell.2023.02.002. 37059069 PMC10107403

[12] Hanahan D , Monje M . Cancer hallmarks intersect with neuroscience in the tumor microenvironment. Cancer Cell. 2023; 41( 3): 573– 80. doi:10.1016/j.ccell.2023.02.012. 36917953 PMC10202656

[13] Venkatesh HS , Johung TB , Caretti V , Noll A , Tang Y , Nagaraja S , et al. Neuronal activity promotes glioma growth through neuroligin-3 secretion. Cell. 2015; 161( 4): 803– 16. doi:10.1016/j.cell.2015.04.012. 25913192 PMC4447122

[14] Venkataramani V , Tanev DI , Strahle C , Studier-Fischer A , Fankhauser L , Kessler T , et al. Glutamatergic synaptic input to glioma cells drives brain tumour progression. Nature. 2019; 573( 7775): 532– 8. doi:10.1038/s41586-019-1564-x. 31534219

[15] Venkatesh HS , Morishita W , Geraghty AC , Silverbush D , Gillespie SM , Arzt M , et al. Electrical and synaptic integration of glioma into neural circuits. Nature. 2019; 573( 7775): 539– 45. doi:10.1038/s41586-019-1563-y. 31534222 PMC7038898

[16] Shi Y , Luo J , Wang X , Zhang Y , Zhu H , Su D , et al. Emerging trends on the correlation between neurotransmitters and tumor progression in the last 20 years: A bibliometric analysis via CiteSpace. Front Oncol. 2022; 12: 800499. doi:10.3389/fonc.2022.800499. 35280754 PMC8907850

[17] Lee JY , Koo BI , Le-Kim TH , Nam Y . Aberrant neuronal firing: A paracrine route to glioblastoma expansion. Cell Commun Signal. 2025; 23( 1): 398. doi:10.1186/s12964-025-02404-8. 40999491 PMC12465965

[18] Matute C , Arellano RO , Conde-Guerri B , Miledi R . mRNA coding for neurotransmitter receptors in a human astrocytoma. Proc Natl Acad Sci U S A. 1992; 89( 8): 3399– 403. doi:10.1073/pnas.89.8.3399. 1348861 PMC48875

[19] Kuhn SA , Mueller U , Hanisch UK , Regenbrecht CRA , Schoenwald I , Brodhun M , et al. Glioblastoma cells express functional cell membrane receptors activated by daily used medical drugs. J Cancer Res Clin Oncol. 2009; 135( 12): 1729– 45. doi:10.1007/s00432-009-0620-6. 19543745 PMC2847174

[20] Belotti Y , Tolomeo S , Yu R , Lim WT , Lim CT . Prognostic neurotransmitter receptors genes are associated with immune response, inflammation and cancer hallmarks in brain tumors. Cancers. 2022; 14( 10): 2544. doi:10.3390/cancers14102544. 35626148 PMC9139273

[21] Weydt P , Möller T , Labrakakis C , Patt S , Kettenmann H . Neuroligand-triggered calcium signalling in cultured human glioma cells. Neurosci Lett. 1997; 228( 2): 91– 4. doi:10.1016/S0304-3940(97)00366-2. 9209106

[22] Taylor KR , Barron T , Hui A , Spitzer A , Yalçin B , Ivec AE , et al. Glioma synapses recruit mechanisms of adaptive plasticity. Nature. 2023; 623( 7986): 366– 74. doi:10.1038/s41586-023-06678-1. 37914930 PMC10632140

[23] Timmer M , Lauer N , Kuhl S , Goldbrunner R . Tmic-48. synaptic proteins bassoon, dlg4, dlg1, magi2, shank1, and Homer1 in glioma. Neuro Oncol. 2023; 25( Suppl 5): v289. doi:10.1093/neuonc/noad179.1114.

[24] Tetzlaff SK , Reyhan E , Layer N , Bengtson CP , Heuer A , Schroers J , et al. Characterizing and targeting glioblastoma neuron-tumor networks with retrograde tracing. Cell. 2025; 188( 2): 390– 411.e36. doi:10.1016/j.cell.2024.11.002. 39644898

[25] Hsieh AL , Ganesh S , Kula T , Irshad M , Ferenczi EA , Wang W , et al. Widespread neuroanatomical integration and distinct electrophysiological properties of glioma-innervating neurons. Proc Natl Acad Sci U S A. 2024; 121( 50): e2417420121. doi:10.1073/pnas.2417420121. 39630872 PMC11648874

[26] Venkataramani V , Schneider M , Giordano FA , Kuner T , Wick W , Herrlinger U , et al. Disconnecting multicellular networks in brain tumours. Nat Rev Cancer. 2022; 22( 8): 481– 91. doi:10.1038/s41568-022-00475-0. 35488036

[27] Sun Y , Wang X , Zhang DY , Zhang Z , Bhattarai JP , Wang Y , et al. Brain-wide neuronal circuit connectome of human glioblastoma. Nature. 2025; 641( 8061): 222– 31. doi:10.1038/s41586-025-08634-7. 39821165 PMC12347542

[28] Liu H , Xia J , Wang T , Li W , Song Y , Tan G . Differentiation of human glioblastoma U87 cells into cholinergic neuron. Neurosci Lett. 2019; 704: 1– 7. doi:10.1016/j.neulet.2019.03.049. 30928478

[29] Wolańczyk M , Hułas-Bigoszewska K , Witusik-Perkowska M , Papierz W , Jaskólski D , Liberski PP , et al. Imperfect oligodendrocytic and neuronal differentiation of glioblastoma cells. Folia Neuropathol. 2010; 48( 1): 27– 34. 20383808

[30] Wakimoto H , Mohapatra G , Kanai R , Curry WT Jr , Yip S , Nitta M , et al. Maintenance of primary tumor phenotype and genotype in glioblastoma stem cells. Neuro Oncol. 2012; 14( 2): 132– 44. doi:10.1093/neuonc/nor195. 22067563 PMC3266381

[31] Assaf S , Bozek D , Luchman HA , Weiss S . Epco-16. disrupting dot1l epigenetic activity reprograms glioblastoma stem cells towards a dopaminergic neuronal-like state. Neuro Oncol. 2023; 25( Suppl 5): v126– 7. doi:10.1093/neuonc/noad179.0479.

[32] Sánchez OF , Rodríguez AV , Velasco-España JM , Murillo LC , Sutachan JJ , Albarracin SL . Role of connexins 30, 36, and 43 in brain tumors, neurodegenerative diseases, and neuroprotection. Cells. 2020; 9( 4): 846. doi:10.3390/cells9040846. 32244528 PMC7226843

[33] Chen P , Wang W , Liu R , Lyu J , Zhang L , Li B , et al. Olfactory sensory experience regulates gliomagenesis via neuronal IGF1. Nature. 2022; 606( 7914): 550– 6. doi:10.1038/s41586-022-04719-9. 35545672

[34] Huang-Hobbs E , Cheng YT , Ko Y , Luna-Figueroa E , Lozzi B , Taylor KR , et al. Remote neuronal activity drives glioma progression through SEMA4F. Nature. 2023; 619( 7971): 844– 50. doi:10.1038/s41586-023-06267-2. 37380778 PMC10840127

[35] Lange F , Hörnschemeyer MF , Kirschstein T . Glutamatergic mechanisms in glioblastoma and tumor-associated epilepsy. Cells. 2021; 10( 5): 1226. doi:10.3390/cells10051226. 34067762 PMC8156732

[36] Chen H , Judkins J , Ghamsari F , Lein P , Horbinski C . Qlif-03. mutant idh1 promotes tumor-associated epilepsy in glioma patients. Neuro Oncol. 2016; 18( Suppl 6): vi156. doi:10.1093/neuonc/now212.649.

[37] Kumaria A , Ashkan K . Novel therapeutic strategies in glioma targeting glutamatergic neurotransmission. Brain Res. 2023; 1818: 148515. doi:10.1016/j.brainres.2023.148515. 37543066

[38] Noch E , Khalili K . Molecular mechanisms of necrosis in glioblastoma: The role of glutamate excitotoxicity. Cancer Biol Ther. 2009; 8( 19): 1791– 7. doi:10.4161/cbt.8.19.9762. 19770591 PMC4503249

[39] Brocke KS , Staufner C , Luksch H , Geiger KD , Stepulak A , Marzahn J , et al. Glutamate receptors in pediatric tumors of the central nervous system. Cancer Biol Ther. 2010; 9( 6): 455– 68. doi:10.4161/cbt.9.6.10898. 20061814

[40] Joghataei MT , Bakhtiarzadeh F , Dehghan S , Ketabforoush AHME , Golab F , Zarbakhsh S , et al. The role of neurotransmitters in glioblastoma multiforme-associated seizures. Int J Dev Neurosci. 2023; 83( 8): 677– 90. doi:10.1002/jdn.10294. 37563091

[41] Pei Z , Lee KC , Khan A , Erisnor G , Wang HY . Pathway analysis of glutamate-mediated, calcium-related signaling in glioma progression. Biochem Pharmacol. 2020; 176: 113814. doi:10.1016/j.bcp.2020.113814. 31954716 PMC8403340

[42] Hausmann D , Hoffmann DC , Venkataramani V , Jung E , Horschitz S , Tetzlaff SK , et al. Autonomous rhythmic activity in glioma networks drives brain tumour growth. Nature. 2023; 613( 7942): 179– 86. doi:10.1038/s41586-022-05520-4. 36517594

[43] Roderick HL , Cook SJ . Ca^2+^ signalling checkpoints in cancer: Remodelling Ca^2+^ for cancer cell proliferation and survival. Nat Rev Cancer. 2008; 8( 5): 361– 75. doi:10.1038/nrc2374. 18432251

[44] Suzuki T , Tsuzuki K , Kameyama K , Kwak S . Recent advances in the study of AMPA receptors. Folia Pharmacol Jpn. 2003; 122( 6): 515– 26. doi:10.1254/fpj.122.515. 14639006

[45] Liu CC , Wu SN , Sze CI . The potential role of NMDA receptor regulating TGF-β/Smad pathway in radiation-induced resistance in glioblastoma multiforme. FASEB J. 2017; 31( S1): 934.7. doi:10.1096/fasebj.31.1_supplement.934.7.

[46] Nishi T , Takahashi M , Ito H , Yoshihama I , Takada E , Mizuguchi J . Participation of bcl-2/bax-α in glutamate-induced apoptosis of human glioblastoma cells. J Neuro Oncol. 1999; 44( 2): 109– 17. doi:10.1023/A:1006310815374. 10619494

[47] D’Alessandro G , Lauro C , Quaglio D , Ghirga F , Botta B , Trettel F , et al. Neuro-signals from gut microbiota: Perspectives for brain glioma. Cancers. 2021; 13( 11): 2810. doi:10.3390/cancers13112810. 34199968 PMC8200200

[48] Sørensen MF , Heimisdóttir SB , Sørensen MD , Mellegaard CS , Wohlleben H , Kristensen BW , et al. High expression of cystine-glutamate antiporter xCT (SLC7A11) is an independent biomarker for epileptic seizures at diagnosis in glioma. J Neurooncol. 2018; 138( 1): 49– 53. doi:10.1007/s11060-018-2785-9. 29404978

[49] Kolb AK , Piccirillo S , Watts C . Sensitizing glioblastoma cells to therapy by targeting the l-glutamate/l-cystine antiporter system X_c_^−^. Neuro Oncol. 2014; 16( Suppl 2): ii34– 5. doi:10.1093/neuonc/nou174.127.

[50] Lo M , Wang YZ , Gout PW . The X_c_^−^ cystine/glutamate antiporter: A potential target for therapy of cancer and other diseases. J Cell Physiol. 2008; 215( 3): 593– 602. doi:10.1002/jcp.21366. 18181196

[51] Musto AE . Glutamate and epilepsy: An insight from temporal lobe epilepsy. In: Glutamate and neuropsychiatric disorders. Berlin/Heidelberg, Germany: Springer; 2022. p. 523– 37. doi:10.1007/978-3-030-87480-3_18.

[52] Scott AJ , Mittal A , Meghdadi B , O’Brien A , Bailleul J , Sravya P , et al. Rewiring of cortical glucose metabolism fuels human brain cancer growth. Nature. 2025; 646( 8084): 413– 22. doi:10.1038/s41586-025-09460-7. 40903569 PMC12507665

[53] Maher EA , Marin-Valencia I , Bachoo RM , Mashimo T , Raisanen J , Hatanpaa KJ , et al. Metabolism of [U-13 C] glucose in human brain tumors *in vivo*. NMR Biomed. 2012; 25( 11): 1234– 44. doi:10.1002/nbm.2794. 22419606 PMC3406255

[54] Luyken C , Blümcke I , Fimmers R , Urbach H , Elger CE , Wiestler OD , et al. The spectrum of long-term epilepsy-associated tumors: Long-term seizure and tumor outcome and neurosurgical aspects. Epilepsia. 2003; 44( 6): 822– 30. doi:10.1046/j.1528-1157.2003.56102.x. 12790896

[55] Chen R , Nishimura MC , Kharbanda S , Peale F , Deng Y , Daemen A , et al. Hominoid-specific enzyme GLUD2 promotes growth of *IDH1^R132H^* glioma. Proc Natl Acad Sci U S A. 2014; 111( 39): 14217– 22. doi:10.1073/pnas.1409653111. 25225364 PMC4191757

[56] Ohno M , Hayashi M , Matsushita Y , Miyakita Y , Takahashi M , Yamazawa E , et al. Ncmp-09. isocitrate dehydrogenase mutations and increased tissue 2-hydroxyglutarate concentration might be related with seizure onset in patients with gliomas. Neuro Oncol. 2018; 20( Suppl 6): vi195. doi:10.1093/neuonc/noy148.809.

[57] Ramadan S , Andronesi OC , Stanwell P , Lin AP , Sorensen AG , Mountford CE . Use of *in vivo* two-dimensional MR spectroscopy to compare the biochemistry of the human brain to that of glioblastoma. Radiology. 2011; 259( 2): 540– 9. doi:10.1148/radiol.11101123. 21357517 PMC3079117

[58] Righi V, Andronesi OC, Mintzopoulos D, Black PM, Tzika AA . High-resolution magic angle spinning magnetic resonance spectroscopy detects glycine as a biomarker in brain tumors. Int J Oncol. 2009; 36( 2): 301– 6. doi:10.3892/ijo_00000500. PMC3715372 20043062

[59] Tzika AA , Astrakas L , Cao H , Mintzopoulos D , Andronesi OC , Mindrinos M , et al. Combination of high-resolution magic angle spinning proton magnetic resonance spectroscopy and microscale genomics to type brain tumor biopsies. Int J Mol Med. 2007; 20( 2): 199– 208. doi:10.3892/ijmm.20.2.199. 17611638

[60] Shevelev OB , Cherkasova OP , Razumov IA , Zavjalov EL . *In vivo* MRS study of long-term effects of traumatic intracranial injection of a culture medium in mice. Vestn VOGiS. 2023; 27( 6): 633– 40. doi:10.18699/VJGB-23-74. PMC10784322 38223456

[61] Deviers A , Ken S , Franceries X , Filleron T , Mogicato G , Lotterie J , et al. Evaluation of lactate as a predictive marker of survival and local response to radiation therapy in patients with GBM. Int J Radiat Oncol. 2012; 84( 3): S271– 2. doi:10.1016/j.ijrobp.2012.07.707.

[62] Tripathy S , Singh S , Banerjee M , Modi DR , Prakash A . Coagulation proteases and neurotransmitters in pathogenicity of glioblastoma multiforme. Int J Neurosci. 2024; 134( 4): 398– 408. doi:10.1080/00207454.2022.2107514. 35896309

[63] Spangelo BL , Horrell S , Goodwin AL , Shroff S , Jarvis WD . Somatostatin and gamma-aminobutyric acid inhibit interleukin-1β-stimulated release of interleukin-6 from rat C6 glioma cells. Neuroimmunomodulation. 2004; 11( 5): 332– 40. doi:10.1159/000079414. 15316244

[64] Barrow R , Poulter JA , Finetti MA , Wilson E , Johnson CA , Stead L . Modulation of GABA neurotransmitter signalling impacts glioblastoma spheroid growth and response to standard treatment. Neuro Oncol. 2022; 24( Suppl 4): iv3– 4. doi:10.1093/neuonc/noac200.013.

[65] Smits A , Jin Z , Elsir T , Pedder H , Nistér M , Alafuzoff I , et al. GABA-A channel subunit expression in human glioma correlates with tumor histology and clinical outcome. PLoS One. 2012; 7( 5): e37041. doi:10.1371/journal.pone.0037041. 22615883 PMC3355166

[66] Barron T , Yalçın B , Su M , Byun YG , Gavish A , Shamardani K , et al. GABAergic neuron-to-glioma synapses in diffuse midline gliomas. Nature. 2025; 639( 8056): 1060– 8. doi:10.1038/s41586-024-08579-3. 39972132 PMC11946904

[67] Chen J , Wang H , Deng C , Fei M . SLC12A5 as a novel potential biomarker of glioblastoma multiforme. Mol Biol Rep. 2023; 50( 5): 4285– 99. doi:10.1007/s11033-023-08371-y. 36917367

[68] El-Habr EA , Dubois LG , Burel-Vandenbos F , Bogeas A , Lipecka J , Turchi L , et al. A driver role for GABA metabolism in controlling stem and proliferative cell state through GHB production in glioma. Acta Neuropathol. 2017; 133( 4): 645– 60. doi:10.1007/s00401-016-1659-5. 28032215 PMC5348560

[69] Thompson EG , Sontheimer H . Acetylcholine receptor activation as a modulator of glioblastoma invasion. Cells. 2019; 8( 10): 1203. doi:10.3390/cells8101203. 31590360 PMC6829263

[70] Cristofaro I , Alessandrini F , Spinello Z , Guerriero C , Fiore M , Caffarelli E , et al. Cross interaction between M2 muscarinic receptor and Notch1/EGFR pathway in human glioblastoma cancer stem cells: Effects on cell cycle progression and survival. Cells. 2020; 9( 3): 657. doi:10.3390/cells9030657. 32182759 PMC7140674

[71] Anand SV , Skorput AG , Gulledge AT , Fox IB , Bonnin DA , Young AL , et al. Abstract 905: Targeting muscarinic acetylcholine receptors in glioma stem like cells. Cancer Res. 2022; 82( 12 Suppl): 905. doi:10.1158/1538-7445.AM2022-905.

[72] Gondarenko E , Mazur D , Masliakova M , Ryabukha Y , Kasheverov I , Utkin Y , et al. Subtype-selective peptide and protein neurotoxic inhibitors of nicotinic acetylcholine receptors enhance proliferation of patient-derived glioblastoma cell lines. Toxins. 2024; 16( 2): 80. doi:10.3390/toxins16020080. 38393158 PMC10891657

[73] Spina R , Voss DM , Asnaghi L , Sloan A , Bar EE . Atracurium Besylate and other neuromuscular blocking agents promote astroglial differentiation and deplete glioblastoma stem cells. Oncotarget. 2016; 7( 1): 459– 72. doi:10.18632/oncotarget.6314. 26575950 PMC4808011

[74] Rump K , Holtkamp C , Bergmann L , Nowak H , Unterberg M , Orlowski J , et al. Midazolam impacts acetyl-And butyrylcholinesterase genes: An epigenetic explanation for postoperative delirium? PLoS One. 2022; 17( 7): e0271119. doi:10.1371/journal.pone.0271119. 35802656 PMC9269431

[75] Caragher SP , Hall RR , Ahsan R , Ahmed AU . Monoamines in glioblastoma: Complex biology with therapeutic potential. Neuro Oncol. 2018; 20( 8): 1014– 25. doi:10.1093/neuonc/nox210. 29126252 PMC6280144

[76] Kato S . Effects of platinum-coexisting dopamine with X-ray irradiation upon human glioblastoma cell proliferation. Hum Cell. 2021; 34( 6): 1653– 61. doi:10.1007/s13577-021-00591-3. 34374034

[77] Marisetty AL , Lu L , Veo BL , Liu B , Coarfa C , Kamal MM , et al. REST-DRD2 mechanism impacts glioblastoma stem cell–mediated tumorigenesis. Neuro Oncol. 2019; 21( 6): 775– 85. doi:10.1093/neuonc/noz030. 30953587 PMC6556851

[78] Li J , Zhu S , Kozono D , Ng K , Futalan D , Shen Y , et al. Genome-wide shRNA screen revealed integrated mitogenic signaling between dopamine receptor D2 (DRD2) and epidermal growth factor receptor (EGFR) in glioblastoma. Oncotarget. 2014; 5( 4): 882– 93. doi:10.18632/oncotarget.1801. 24658464 PMC4011590

[79] Li J , Zhu S , Kozono D , Futulan D , Gonda D , Kushwaha D , et al. Abstract 4366: ShRNA-based cellular proliferation signaling analysis revealed DRD2 as a novel therapeutic target for glioblastoma. Cancer Res. 2013; 73( 8 Suppl): 4366. doi:10.1158/1538-7445.AM2013-4366.

[80] Caragher SP , Park CH , Atashi F , Baisiwala S , Ahmed AU . Abstract 2888: Dopamine signaling and therapeutic resistance in GBM. Cancer Res. 2017; 77( 13 Suppl): 2888. doi:10.1158/1538-7445.AM2017-2888.

[81] Caragher SP , Shireman JM , Huang M , Miska J , Atashi F , Baisiwala S , et al. Activation of dopamine receptor 2 prompts transcriptomic and metabolic plasticity in glioblastoma. J Neurosci. 2019; 39( 11): 1982– 93. doi:10.1523/JNEUROSCI.1589-18.2018. 30651332 PMC6507082

[82] Caragher SP , Park CH , Atashi F , Guo D , Lesniak MS , James C , et al. P01.02 The role of dopamine signaling in GBM recurrence. Neuro Oncol. 2017; 19( Suppl 3): iii23. doi:10.1093/neuonc/nox036.078.

[83] Wang Y , Wang X , Wang K , Qi J , Zhang Y , Wang X , et al. Chronic stress accelerates glioblastoma progression via DRD2/ERK/β-catenin axis and Dopamine/ERK/TH positive feedback loop. J Exp Clin Cancer Res. 2023; 42( 1): 161. doi:10.1186/s13046-023-02728-8. 37415171 PMC10327168

[84] Lonjon M , Quentien MH , Risso JJ , Michiels JF , Carre E , Rostain JC , et al. Alteration of striatal dopaminergic function induced by glioma development: A microdialysis and immunohistological study in the rat striatum. Neurosci Lett. 2004; 354( 2): 131– 4. doi:10.1016/j.neulet.2003.10.005. 14698456

[85] You F , Zhang C , Liu X , Ji D , Zhang T , Yu R , et al. Drug repositioning: Using psychotropic drugs for the treatment of glioma. Cancer Lett. 2022; 527: 140– 9. doi:10.1016/j.canlet.2021.12.014. 34923043

[86] Weissenrieder JS , Reed JL , Green MV , Moldovan GL , Koubek EJ , Neighbors JD , et al. The dopamine D2 receptor contributes to the spheroid formation behavior of U87 glioblastoma cells. Pharmacology. 2020; 105( 1–2): 19– 27. doi:10.1159/000502562. 31645049 PMC10777736

[87] Ware M , Pontual LL , Yu C , Kushida M , Rastegar N , Dolma S , et al. Cnsc-29. investigating the influence of dopaminergic activity on the glioblastoma niche. Neuro Oncol. 2022; 24( Suppl 7): vii28. doi:10.1093/neuonc/noac209.110.

[88] Sturzu A , Sheikh S , Klose U , Echner H , Kalbacher H , Deeg M , et al. Using the neurotransmitter serotonin to target imaging agents to glioblastoma cells. Investig New Drugs. 2012; 30( 6): 2141– 7. doi:10.1007/s10637-011-9781-7. 22212740

[89] Ting J , Sherman P , Yokota S , Moore T , Basta P . Neurotransmitter modulation of the human class II gene DRα on multiforme glioblastoma cell lines a molecular analysis. Ann N Y Acad Sci. 1988; 540( 1): 477– 8. doi:10.1111/j.1749-6632.1988.tb27141.x. 2849898

[90] Sarrouilhe D , Defamie N , Mesnil M . Is the exposome involved in brain disorders through the serotoninergic system? Biomedicines. 2021; 9( 10): 1351. doi:10.3390/biomedicines9101351. 34680468 PMC8533279

[91] Romero-Reyes J , Vázquez-Martínez ER , Molina-Hernández A , Silva CC , Hernández-Montes G , Peña-Gutiérrez KM , et al. The molecular footprint of the serotoninergic system in human glioblastoma cells. J Mol Histol. 2025; 56( 6): 371. doi:10.1007/s10735-025-10647-5. 41219608

[92] Zhong J , Shan W , Zuo Z . Norepinephrine inhibits migration and invasion of human glioblastoma cell cultures possibly via MMP-11 inhibition. Brain Res. 2021; 1756: 147280. doi:10.1016/j.brainres.2021.147280. 33515535 PMC7904089

[93] Kraboth Z , Kajtár B , Gálik B , Gyenesei A , Miseta A , Kalman B . Involvement of the catecholamine pathway in glioblastoma development. Cells. 2021; 10( 3): 549. doi:10.3390/cells10030549. 33806345 PMC7998903

[94] Krabóth Z , Tompa M , Urbán P , Gálik B , Kajtár B , Gyenesei A , et al. Glioblastoma epigenomics discloses a complex biology and potential therapeutic targets. Ideggyogy Sz. 2024; 77( 1–2): 27– 37. doi:10.18071/isz.77.0027. 38321856

[95] Shih JC . Monoamine oxidase isoenzymes: Genes, functions and targets for behavior and cancer therapy. J Neural Transm. 2018; 125( 11): 1553– 66. doi:10.1007/s00702-018-1927-8. 30259128 PMC6245662

[96] Li PC , Chen SY , Xiangfei D , Mao C , Wu CH , Shih JC . PAMs inhibits monoamine oxidase a activity and reduces glioma tumor growth, a potential adjuvant treatment for glioma. BMC Complementary Med Ther. 2020; 20( 1): 252. doi:10.1186/s12906-020-03041-z. PMC7429690 32799864

[97] Ou XM , Chen K , Shih JC . Glucocorticoid and androgen activation of monoamine oxidase a is regulated differently by R1 and Sp1. J Biol Chem. 2006; 281( 30): 21512– 25. doi:10.1074/jbc.M600250200. 16728402

[98] Afshari AR , Motamed-Sanaye A , Sabri H , Soltani A , Karkon-Shayan S , Radvar S , et al. Neurokinin-1 receptor (NK-1R) antagonists: Potential targets in the treatment of glioblastoma multiforme. Curr Med Chem. 2021; 28( 24): 4877– 92. doi:10.2174/0929867328666210113165805. 33441062

[99] Grouzmann E , Meyer C , Bürki E , Brunner H . Neuropeptide Y Y2 receptor signalling mechanisms in the human glioblastoma cell line LN319. Peptides. 2001; 22( 3): 379– 86. doi:10.1016/S0196-9781(01)00344-8. 11287092

[100] Jóźwiak-Bębenista M , Kowalczyk E . Neuroleptic drugs and PACAP differentially affect the mRNA expression of genes encoding PAC1/VPAC type receptors. Neurochem Res. 2017; 42( 4): 943– 52. doi:10.1007/s11064-016-2127-2. 27900577 PMC5375968

[101] Chu J , Lee L , Siu F , Chow B . The secretin/pituitary adenylate cyclase-activating polypeptide/vasoactive intestinal polypeptide superfamily in the central nervous system. Cent Nerv Syst Agents Med Chem. 2006; 6( 1): 27– 57. doi:10.2174/187152406776056546.

[102] García SI , Porto PI , Martinez VN , Alvarez AL , Finkielman S , Pirola CJ . Expression of TRH and TRH-like peptides in a human glioblastoma-astrocytoma cell line (U-373-MG). J Endocrinol. 2000; 166( 3): 697– 703. doi:10.1677/joe.0.1660697. 10974663

[103] Strong AD , Indart MC , Hill NR , Daniels RL . GL261 glioma tumor cells respond to ATP with an intracellular calcium rise and glutamate release. Mol Cell Biochem. 2018; 446( 1): 53– 62. doi:10.1007/s11010-018-3272-5. 29318454 PMC6037622

[104] Debom GN , Rubenich DS , Braganhol E . Adenosinergic signaling as a key modulator of the glioma microenvironment and reactive astrocytes. Front Neurosci. 2022; 15: 648476. doi:10.3389/fnins.2021.648476. 35069091 PMC8766410

[105] Gugnani K , Rondon-Ortiz AN , Juarez-Valdez A , Pardes-Quiroz JM , Pino-Figueroa A . Antiproliferative activity of endocannabinoids in U87-MG glioblastoma cells. FASEB J. 2017; 31( S1): 996.16. doi:10.1096/fasebj.31.1_supplement.996.16.

[106] Movsesyan VA , Stoica BA , Yakovlev AG , Knoblach SM , Lea PM 4th , Cernak I , et al. Anandamide-induced cell death in primary neuronal cultures: Role of calpain and caspase pathways. Cell Death Differ. 2004; 11( 10): 1121– 32. doi:10.1038/sj.cdd.4401442. 15375383

[107] Mathew B , Harilal S , Musa A , Kumar R , Parambi DGT , Jose J , et al. An agathokakological tale of Δ9-THC: Exploration of possible biological targets. Curr Drug Targets. 2021; 22( 7): 823– 34. doi:10.2174/1389450121666201001123515. 33001012

[108] Shoji K , Mariotto S , Ciampa AR , Suzuki H . Regulation of serine racemase activity by d-serine and nitric oxide in human glioblastoma cells. Neurosci Lett. 2006; 392( 1–2): 75– 8. doi:10.1016/j.neulet.2005.08.063. 16182447

[109] Sawicka MM , Sawicki K , Łysoń T , Polityńska B , Miltyk W . Proline metabolism in malignant gliomas: A systematic literature review. Cancers. 2022; 14( 8): 2030. doi:10.3390/cancers14082030. 35454935 PMC9027994

[110] Lam-Himlin D , Espey MG , Perry G , Smith MA , Castellani RJ . Malignant glioma progression and nitric oxide. Neurochem Int. 2006; 49( 8): 764– 8. doi:10.1016/j.neuint.2006.07.001. 16971023

[111] Irshad K , Singh S , Ali Ahmad K , Aghoghovwia B , Alnassiry S , Lang F , et al. Cnsc-18. differential effects of neurotransmitters on the growth of glioblastoma subtypes. Neuro Oncol. 2023; 25( Suppl 5): v26. doi:10.1093/neuonc/noad179.0102.

[112] Romero-Reyes J , Vázquez-Martínez ER , Silva CC , Molina-Hernández A , Díaz NF , Camacho-Arroyo I . Navigating glioblastoma complexity: The interplay of neurotransmitters and chromatin. Mol Biol Rep. 2024; 51( 1): 912. doi:10.1007/s11033-024-09853-3. 39153092 PMC11330389

[113] Mondal A , Saha S , Ghosh A , Lathia JD , Das Sarma J . Connexin43 functions as a non-canonical phenotypic stability factor in promoting hybrid Epithelial/Mesenchymal phenotype in glioblastoma cells. Transl Oncol. 2025; 59: 102463. doi:10.1016/j.tranon.2025.102463. 40591997 PMC12269574

[114] Mastall M , Roth P , Bink A , Fischer Maranta A , Läubli H , Hottinger AF , et al. A phase Ib/II randomized, open-label drug repurposing trial of glutamate signaling inhibitors in combination with chemoradiotherapy in patients with newly diagnosed glioblastoma: The GLUGLIO trial protocol. BMC Cancer. 2024; 24( 1): 82. doi:10.1186/s12885-023-11797-z. 38225589 PMC10789019

[115] Wirsching HG , Roth P , Fischer A , Hottinger A , Hundsberger T , Migliorini D , et al. Rtid-04. Gluglio—A phase ib/ii randomized drug repurposing trial of glutamate signaling inhibitors in combination with chemoradiotherapy in patients with newly diagnosed glioblastoma (nct05664464). Neuro Oncol. 2023; 25( Suppl 5): v259. doi:10.1093/neuonc/noad179.0995. PMC10789019 38225589

[116] Tazik S , Johnson S , Lu D , Johnson C , Youdim MBH , Stockmeier CA , et al. Comparative neuroprotective effects of rasagiline and aminoindan with selegiline on dexamethasone-induced brain cell apoptosis. Neurotox Res. 2009; 15( 3): 284– 90. doi:10.1007/s12640-009-9030-4. 19384601 PMC2754804

[117] Abadi B , Shahsavani Y , Faramarzpour M , Rezaei N , Rahimi HR . Antidepressants with anti-tumor potential in treating glioblastoma: A narrative review. Fundam Clin Pharmacol. 2022; 36( 1): 35– 48. doi:10.1111/fcp.12712. 34212424

[118] Sah DWY . Therapeutic potential of RNA interference for neurological disorders. Life Sci. 2006; 79( 19): 1773– 80. doi:10.1016/j.lfs.2006.06.011. 16815477

[119] McConnell EM , Holahan MR , Derosa MC . Aptamers as promising molecular recognition elements for diagnostics and therapeutics in the central nervous system. Nucleic Acid Ther. 2014; 24( 6): 388– 404. doi:10.1089/nat.2014.0492. 25296265

[120] Sahu JK , Mishra AK . Tools in the design of therapeutic drugs for CNS disorders: An up-to-date review. Curr Mol Pharmacol. 2018; 11( 4): 270– 8. doi:10.2174/1874467211666180821101158. 30129422

[121] Neftel C , Laffy J , Filbin MG , Hara T , Shore ME , Rahme GJ , et al. An integrative model of cellular states, plasticity, and genetics for glioblastoma. Cell. 2019; 178( 4): 835– 49.e21. doi:10.1016/j.cell.2019.06.024. 31327527 PMC6703186

[122] Paixao Becker A , Hlavin Bell E , Haque SJ , McElroy J , Fleming J , Han C , et al. Path-19. tumor heterogeneity in gliomas: A pilot study of histopathology-associated proteome profiles assessed by liquid chromatography tandem mass spectrometry of ffpe samples. Neuro Oncol. 2019; 21( Suppl 6): vi147. doi:10.1093/neuonc/noz175.615.

